# Immunological Effects of Diesel Particles in a Murine Model of Healthy Mice

**DOI:** 10.3390/toxics12080530

**Published:** 2024-07-23

**Authors:** David Soler-Segovia, Miquel de Homdedeu, Silvia Sánchez-Díez, Christian Romero-Mesones, David Espejo, Fopke Marain, Jeroen Vanoirbeek, Xavier Munoz, María-Jesús Cruz

**Affiliations:** 1 Pulmonology Service, Hospital Universitari Vall d’Hebron, 08035 Barcelona, Spain; david.soler@vhir.org (D.S.-S.); mhomdedeu@bst.cat (M.d.H.); 93sanchezsilvia@gmail.com (S.S.-D.); christian.romero@vhir.org (C.R.-M.); david.espejo@vhir.org (D.E.); mj.cruz@vhir.org (M.-J.C.); 2 CIBER Enfermedades Respiratorias (CibeRes), 08035 Barcelona, Spain; 3 Medicine Department, Universitat Autònoma de Barcelona, 08035 Barcelona, Spain; 4 Laboratory of Respiratory Diseases and Thoracic Surgery, Department of Chronic Diseases and Metabolism, KU Leuven, 3000 Leuven, Belgium; fopke.marain@kuleuven.be; 5 Centre of Environment and Health, Department of Public Health and Primary Care, KU Leuven, 3000 Leuven, Belgium; jeroen.vanoirbeek@kuleuven.be; 6 Department of Cell Biology, Physiology and Immunology, Universitat Autònoma de Barcelona, 08035 Barcelona, Spain

**Keywords:** pollution, lung inflammation, macrophages, exposure, monocyte

## Abstract

**Introduction:** Exposure to environmental pollutants such as diesel exhaust particles (DEP) increases the risk of respiratory disease exacerbation. However, the possible effects of these particles on the general population remain poorly understood. The present study aimed to assess the immunomodulatory and inflammatory effects of the inhalation of DEP in a model of healthy mice undergoing short-, mid- and long-term exposure. **Materials and Methods:** BALB/c ByJ mice were randomly divided into five experimental groups. The control group received three intranasal instillations of saline over 8 days while the other four groups received intranasal instillations of 150 µg of DEP 3 days per week for 8, 17, 26, and 53 days. Lung function assessment and flow cytometry were performed. **Results:** In lung tissue, intranasal exposure to DEP decreased total monocytes (*p* < 0.015 in all groups). At 26 days, a reduction in inflammatory monocytes and an increase in resident monocytes were observed, *p* = 0.001 and 0.0001, respectively. Eosinophils and neutrophils decreased at 26 days (*p* = 0.017 and *p* = 0.041, respectively). The intranasal challenges of DEP increased the total population of dendritic cells (DC) at 26 and 53 days (*p* = 0.017 and *p* = 0.022, respectively) and decreased the total and alveolar populations of macrophages (*p* < 0.003 for all groups compared to control), while interstitial macrophage populations increased over the time period (*p* = 0.0001 for all groups compared to control). **Conclusions:** Continuous DEP exposure triggers immune mechanisms that predispose healthy individuals to a pro-inflammatory and hyper-reactive microenvironment. This mouse model provides evidence of the capacity of DEP to increase DC, interstitial macrophages, and resident monocytes.

## 1. Introduction

The increase in air pollution over the last decade is one of the most important drivers of the development of respiratory diseases [1,2]. Diesel exhaust particles (DEP) are the major component of the solid fraction of air pollution, mainly due to the extended use of diesel engines worldwide and the presence of industrial sources. DEP present a complex structure comprising a central insoluble carbon core and adsorbed fossil fuel byproducts, such as metals and polycyclic aromatic hydrocarbons (PAH) [3]. These particles have severely harmful impacts on the lungs via a range of pathways including tissue damage due to oxidative stress, triggering inflammatory and immunological responses, as well as via epigenetic dysregulation [4,5,6,7].

Epidemiological studies have been performed to characterize the impacts of traffic related pollution in different respiratory diseases [8,9,10]. However, their impact on healthy individuals remains poorly investigated. In fact, epidemiological studies of early childhood exposure to pollution are the main source of information on the effects of DEP and/or traffic related pollution on healthy individuals. The major conclusion of these studies is that exposure to air pollution increases the risk of developing respiratory diseases such as asthma and allergy [11,12,13,14]. In adults, recent studies assessed how an isolated exposure to air pollution and, specifically to DEPs, is able to produce pro-inflammatory markers and reduce the lung function parameters in healthy individuals [15,16,17,18]. However, the immunological pathways affected during chronic exposure at a more environmentally equivalent DEP concentration have not been studied in depth.

In order to fill this gap in our knowledge, the present study aimed to assess the immunomodulatory and inflammatory effects of the inhalation of DEP in a model of healthy mice undergoing short-, mid- and long-term exposure.

## 2. Materials and Methods

### 2.1. Animals

Six-week-old female BALB/c ByJ mice, weighing 16–19 g, were obtained from Charles River (Lyon, France). The mice were housed in individually ventilated cages in a conventional animal house with 12-h dark/light cycles, and received slightly acidified water and pelleted food (Teklad, 2014; Harlan Laboratories, Indianapolis, IN, USA) ad libitum. One group of mice was used to analyze airway hyperresponsiveness and lung mechanics. A second group was used to analyze the leukocyte pattern in total lung tissue by flow cytometry. All experimental procedures were approved by the local Ethical Committee for Animal Experiments of the Vall d’Hebron Research Institute (CEEA 71/20).

### 2.2. Diesel Exhaust Particles (DEP)

Diesel exhaust particles (Standard Reference Material (SRM) 2975) were purchased from the National Institute of Standards and Technology (NIST) (Gaithersburg, MD, USA). The reported mean diameter of the particles was 11.2 ± 0.1 μm by area distribution, mean diameter by number distribution of 1.62 ± 0.01 μm, ranging from >110 μm to ultrafine particles, and the surface area was 0.538 ± 0.006 m^2^·cm^−3^. Certified mass fraction values for the most abundant PAHs, such as 2-methylphenanthrene, 1-methylphenanthrene, triphenylene, dibenzo[b,k]fluoranthene, 3-nitrofluoranthene, 1-nitropyrene, 7-nitrobenz[a]anthracene, and 6-nitrochrysene, were 2.22 ± 0.21, 0.923 ± 0.057, 5.32 ± 0.24, 2.54 ± 0.08, 3.80 ± 0.24, 35.2 ± 2.2, 3.57 ± 0.32, and 2.45 ± 0.33 mg·kg^−1^, respectively. All experiments were carried out with the same batch.

### 2.3. Experimental Design

**Figure 1:**
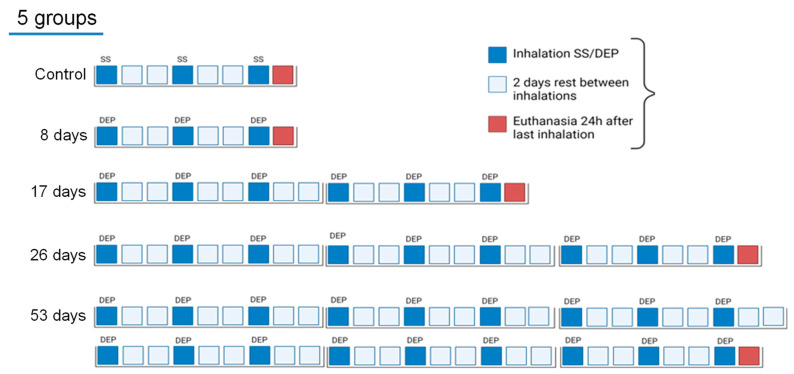
Schematic diagram of the experimental design and the experimental groups. Groups were a control group which inhaled 40 μL of saline (SS) for 8 days, and four experimental groups which inhaled 150 µg of DEP diluted in 40 μL of SS over 8 days, 17 days, 26 days, and 53 days, respectively.

The first set of BALB/c ByJ mice were randomly divided into five experimental groups (five mice per group) to assess airway hyperresponsiveness, lung mechanics, and bronchoalveolar lavage (BAL). The second set of BALB/c ByJ mice were randomly divided into five experimental groups (eight to twelve mice per group) to assess the relative amounts of eosinophils; neutrophils; T and B cells; natural killer (NK) cells; total CD11b+Ly6C+, CD11b+Ly6C−, CD11b-Ly6C+, and CD11b-Ly6C− dendritic cells (DC); total alveolar and interstitial macrophages; and total inflammatory and resident monocytes from lung tissue immune cells by flow cytometry. Mice received a single intranasal instillation under light anesthesia with isoflurane (Forane, Abbott Laboratories, Madrid, Spain), of either 40 μL of saline or 150 µg of DEP diluted in 40 μL of saline, 3 days per week. The experimental model was the same for both batches of mice (Figure 1). The animals were euthanized 24 h after the last inhalation (8, 17, 26, and 53 days, respectively) (controls 8 days).

### 2.4. Lung Function Assessment

In the first group of mice, 24 h after the last intranasal instillation, lung mechanics were assessed invasively using the FlexiVent FX system (SCIREQ Inc., Montreal, QC, Canada). The equipment contains an FX1 module, a negative pressure forced expiration (NPFE) for mice. Mice were deeply anesthetized by an intraperitoneal injection of pentobarbital sodium (70 mg kg-body weight) (Nembutal, Abbott Laboratories, Madrid, Spain); the trachea was exposed, tracheotomized, and connected to the ventilator by an 18-gauge metal cannula. Mice were ventilated with a tidal volume of 10 mL kg^−1^, a maximum inflation pressure (Pmax) of 30 cmH_2_O, a positive end expiratory pressure (PEEP) of 3 cmH_2_O, and a frequency of 150 min^−1^.

First, lungs were inflated totally with two deep inflations at 30 cmH_2_O for lung volume standardization. Then, after 3 s for lung stabilization, inspiratory capacity (IC) was measured. Quick-prime 3 (QP3) was used to measure input impedances (Zrs) over a range of 1–20.5 Hz and then central airway resistance (Rn), tissue elastance (H), and tissue damping (G) were obtained. Finally, an NPFE maneuver was performed to obtain FV loops and the FE-related parameters. Lungs were inflated at +30 cmH_2_O (1.2 s) and then exposed to a negative pressure of −55 cmH_2_O, inducing the negative expiratory pressure gradient. From this, forced expiratory volume at 0.1 s (FEV0.1), forced vital capacity (FVC), and peak expiratory flows (PEF) were obtained and FEV0.1/FVC at 0.1 s, referred to as the Tiffeneau index in humans, was calculated.

### 2.5. Bronchoalveolar Lavage

Bronchoalveolar lavage (BAL) was performed via surgical tracheostomy with an 18-gauge metal needle, and lungs were washed three times in situ with 0.7 mL of sterile saline and pooled. Cells were counted, and pooled fluid was centrifuged (1000× *g*, 10 min). For differential counting, cells (100,000 cells mL^−1^) were spun (450 rpm 6min) (Cytospin 3, Shandon, TechGen, Zellik, Belgium) onto microscope slides, air dried, and stained. For each sample, 300 cells were counted for the number of macrophages, eosinophils, neutrophils, and lymphocytes.

### 2.6. DEP-Uptake Analysis

In BAL cytospin slides, the high contrast of stain and DEP allowed visual discrimination of the particles and cellular contents. First, 100 macrophages were counted and classified as loaded (any % of cytoplasmic coverage) and non-loaded. Then from the loaded group, the % of area covered was measured using ImageJ software 1.54 (NIH, Bethesda, MD, USA), according to the method described [19,20]. Fifty macrophages per mouse were delineated manually and, using ImageJ, the % of area covered by DEP was calculated.

### 2.7. Flow Cytometry

The second set of mice were treated with 50 µL of 1000 U·ml^−1^ s.c. heparin and deeply anesthetized with isofluorane (Forane, Abbott Laboratories, Madrid, Spain) 24 h after the last intranasal instillation and prior to euthanasia. The following cytometry procedure was applied, as described elsewhere [21]. Briefly, after total blood extraction and bronchoalveolar lavage, each mouse was perfused with ice-cold PBS through the heart’s right atrium, and the lungs were inflated with digestion solution (DS) containing collagenase A (Roche, Basel, Switzerland) and DNaseI (Roche), tied with sutures and preserved on ice. All five lobes were carefully removed, minced, digested with DS, filtered through a 70 µm cell strainer (Corning, NY, USA), treated with ACK lysing buffer (Gibco, Carlsbad, CA, USA), and washed twice.

After counting cells with a hemocytometer, 10^6^ cells were stained with BD Horizon Fixable Viability Stain 510 (FVS510), washed twice, and incubated with a blocking solution containing BD Horizon Brilliant Stain Buffer and purified rat anti-mouse CD16/CD32 (Mouse BD Fc Block). After this incubation, BD Horizon APC-R700 rat anti-CD11b, BD Horizon BV786 hamster anti-mouse CD11c, BD Pharmingen APC-Cy7 rat anti-mouse CD45, BD Pharmingen BV605 rat anti-mouse I-A/I-E, BD Pharmingen PE rat anti-mouse CD24, BD OptiBuild BV650 rat anti-mouse Ly-6G, Biolegend Brilliant Violet 421 anti-mouse CD64, and eBioscience PerCP-Cyanine5.5 Ly-6C monoclonal antibodies were added. Compensation, unstained and fluorescence minus one (FMOs) controls were used to set up the panel and reanalyzed with each stained sample. Data were acquired by an LSR Fortessa (BD Biosciences, San Jose, CA, USA) cell analyzer using FACSDiva software (V9.0) (BD Biosciences) and analyzed using FlowJo software (v10.10, TreeStar, Ashland, OR, USA).

### 2.8. Data Analysis

No statistical procedures were applied to pre-determine the group sizes, but our figures were similar to those generally applied in the field. Data are shown as means and standard deviations (SD) or as individual data and group medians. Parametric and nonparametric statistics were performed according to data distribution, as determined by the Shapiro–Wilk normality test. Comparisons were performed using the Kruskal–Wallis test followed by a Dunn’s multiple comparisons test and one-way ANOVA and then followed by Tukey’s post hoc test. A *p* level < 0.05 was considered to be significant. Statistical analysis was performed with SPSS statistical software system version 20 (SPSS, Inc., Chicago, IL, USA) and graphs were generated using GraphPad software (GraphPad Prism 6.01, Graphpad Software Inc., San Diego, IL, USA).

## 3. Results

### 3.1. Lung Function Assessment

**Figure 2:**
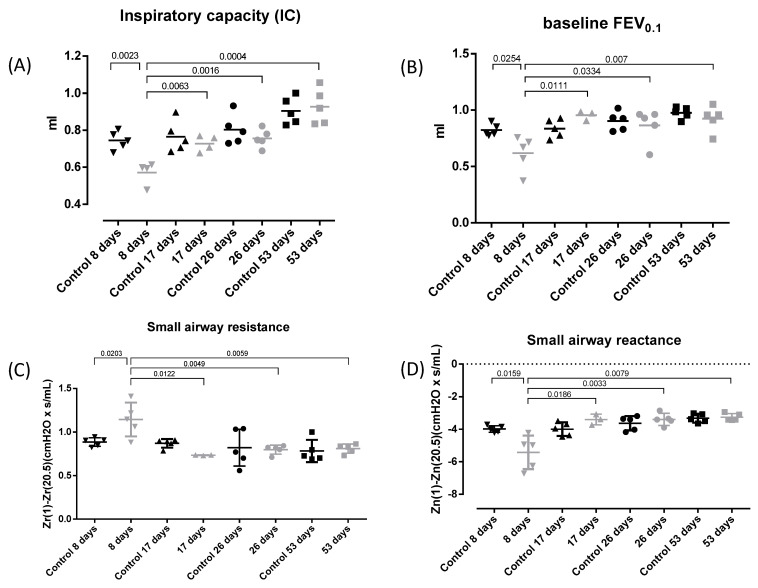
Lung function assessment. Experimental groups are the same as in Figure 1 with a control group added for each time point. Individual and mean values for IC (**A**), FEV_0_._1_ (**B**), small airway resistance (**C**), and small airway reactance (**D**) at baseline. Mean IC (mL): 0.7445; 0.5708; 0.7645; 0.7278; 0.8032; 0.7561; 0.9044, and 0.927 for Control 8 days, 8 days, Control 17 days, 17 days, Control 26 days, 26 days, Control 53 days and 53-day groups, respectively. Mean FEV_0_._1_ (mL): 0.8233; 0.6179; 0.8359; 0.9546; 0.903; 0.8643; 0.9756; and 0.9244 for Control 8 days, 8 days, Control 17 days, 17 days, Control 26 days, 26 days, Control 53 days and 53-day groups, respectively. Mean small airway resistance (cmH_2_O × s/mL): 0.8859; 1.145; 0.8707; 0.7343; 0.8204; 0.7987; 0.7826, and 0.8102 for Control 8 days, 8 days, Control 17 days, 17 days, Control 26 days, 26 days, Control 53 days and 53-day groups, respectively. Mean small airway reactance (cmH_2_O × s/mL): −3.985; −5.418; −3.993; −3.401; −3.63; −3.394; −3.323; and −3.254 for Control 8 days, 8 days, Control 17 days, 17 days, Control 26 days, 26 days, Control 53 days and 53-day groups, respectively. Some lung function measurements were excluded because they did not meet the quality criteria.

Lung function assessment was performed following the parameters described above. This analysis allows us to describe functional and tissue parameters at baseline. Figure 2 shows inspiratory capacity (IC) (A), forced expiratory volume at 0.1 s (FEV0.1) (B), small airway resistance (C), and small airway reactance (D). Due to the substantial influence of weight on the outcomes of respiratory functional tests, it was decided to use age-matched controls in order to mitigate this confounding variable. To this end, additional groups were incorporated, namely 17-day, 26-day, and 56-day controls. However, weight did not significantly impact other procedures within the current experiment. Therefore, in accordance with the principle of refinement, these groups were solely dedicated to the assessment of lung function.

A reduction in inspiratory capacity (IC) was observed in the 8-day experimental group compared to the 8-day control group (*p* = 0.0023) and to the rest of the groups (17-, 26-, and 53-day groups, *p* = 0.0063, *p* = 0.0016 and *p* = 0.0004, respectively). Similarly, a significant reduction in FEV0.1 at baseline was observed in the 8-day group compared to the 8-day control group (*p* = 0.0254) and also compared to the rest of the groups (17-, 26- and 53-day groups, *p* = 0.0111, *p* = 0.0334, and *p* = 0.007, respectively).

As regards tissue parameters—tissue damping (G) and elastance (H)—(Supplementary Figures S1A and S1B, respectively), the same data structure was observed as in functional parameters. The 8-day experimental group presented increased G and H compared to the 8-day control group (*p* = 0.0047 and *p* = 0.0057, respectively) and to the rest of the groups (17-, 26-, and 53-days groups, *p* = 0.0155, *p* = 0.0047 and *p* = 0.0047, respectively, for G; and *p* = 0.0415, *p* = 0.0088 and *p* = 0.0058, respectively for H).

To establish whether the changes described above were more pronounced in the smaller or upper airways, small airway resistance and reactance were calculated by subtracting the highest frequency impedance Z (20.5 Hz) from the lowest frequency impedance Z (1 Hz) in accordance with the literature [22]. The 8-day experimental group presented increased resistance and decreased reactance compared to the 8-day control group (*p* = 0.0203 and *p* = 0.0159, respectively). This increased resistance and decreased reactance were also observed compared to the other groups (17-, 26-, and 53-day groups, *p* = 0.0122, *p* = 0.0049, and *p* = 0.0059, respectively, for small airway resistance; and *p* = 0.0186, *p* = 0.0033, and *p* = 0.0079, respectively, for small airway reactance).

### 3.2. Bronchoalveolar Lavage

**Figure 3:**
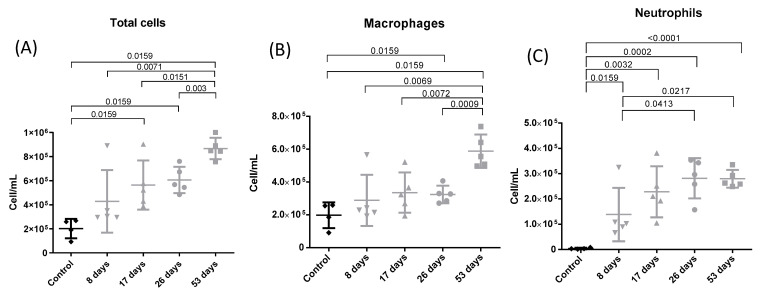
Leukocyte levels from differential counting in bronchoalveolar lavage (BAL). Experimental groups are the same as in Figure 1. Cell/mL of total cells (**A**), cell/mL of macrophages (**B**), and cell/mL of neutrophils (**C**). Mean total cells (cell/mL): 201,500; 426,800; 563,200; 605,600, and 867,400 for control, 8, 17-, 26-, and 53-day groups, respectively. Mean macrophages (cell/mL): 197,634; 288,417; 335,045; 323,883; and 587,744 for control, 8, 17-, 26-, and 53-day groups, respectively. Mean neutrophils (cell/mL): 3866; 138,384; 228,156; 281,718; and 279,657 for control, 8, 17-, 26-, and 53-day groups, respectively.

Figure 3 shows individual and median values of total cells (A), macrophages (B), neutrophils (C), and lymphocytes (D) in bronchoalveolar lavage (BAL) 24 h after the last intranasal inhalation. A statistically significant, progressive increase in the total cell number (A) was observed over the course of the experiment from 17 days. A significant increase in macrophages (B) was observed in the 53-day group compared to control, 8-, 17-, and 26-day groups (*p* = 0.0159, *p* = 0.0069, *p* = 0.0072, and *p* = 0.009, respectively). As for neutrophils (C), a significant increase was observed for all experimental groups compared to controls (*p* = 0.0159, *p* = 0.0032, *p* = 0.0002, and *p* ≤ 0.001 for 8-, 17-, 26-, and 53-days groups, respectively). This increase was also observed between the 8- and 26-day groups (*p* = 0.0413) and the 8- and 53-day groups (*p* = 0.0217).

### 3.3. DEP-Uptake Analysis

**Figure 4:**
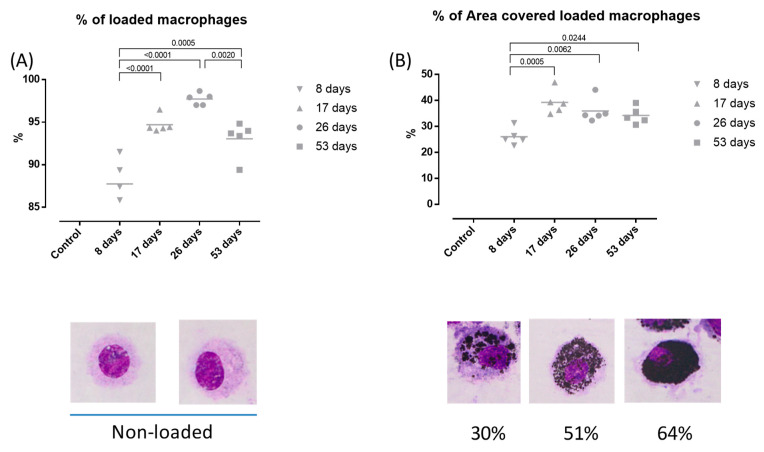
Deep uptake analysis in bronchoalveolar lavage (BAL). Experimental groups are the same as in Figure 1. % of loaded macrophages (**A**) and % of area covered in loaded macrophages (**B**). Mean % of loaded macrophages: 0; 87.74; 94.7; 97.72; and 93.05 for control, 8, 17-, 26-, and 53-day groups, respectively. Mean % of area covered in loaded macrophages: 0; 26.03; 39.24; 35.76; and 34.23 for control, 8, 17-, 26-, and 53-day groups, respectively. Slides photographed at 100× magnification.

Figure 4 displays the % of DEP loaded macrophages (A). No loaded macrophages were observed in the control group. During the exposure, % of loaded macrophages increased when compared to the 8-day group (*p* < 0.0001, *p* < 0.0001, and *p* = 0.005 for 17-, 26-, and 53-day groups, respectively), reaching the maximum at 26 days. However, there was also a reduction in the 53-day group compared to the 26-day group (*p* = 0.0020).

Figure 4 also displays the % of area covered in the loaded macrophages (B). A significant increase was observed in the 17-, 26-, and 53-day groups compared to the 8-day group (*p* = 0.0005, *p* = 0.0062 and *p* = 0.0244, respectively), reaching its peak in the 17-day group. Despite the downward trend, no significant differences were observed for the mid- and long-term DEP exposure groups.

### 3.4. Flow Cytometry

Using the panel described above, we were able to discriminate and quantify the relative number of eosinophils; neutrophils; T and B cells; natural killer (NK) cells; total dendritic cells (DC); total alveolar and interstitial macrophages; and total, inflammatory and resident monocytes from lung tissue immune cells (Figures S2 and S3).

**Figure 5:**
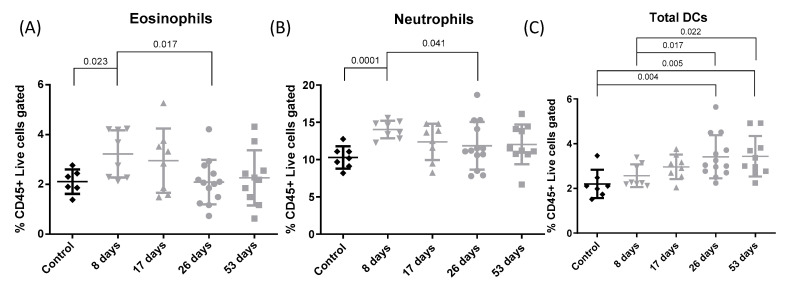
Lung eosinophils, neutrophils and DC. Experimental groups are the same as in Figure 1. Individual and mean values of total eosinophils (**A**), neutrophils (**B**), and DC (**C**) from flow cytometry analysis. Mean total eosinophils (% CD45+ live cells gated): 2.111; 3.224; 2.952; 2.091, and 2.262 for control, 8, 17-, 26-, and 53-day groups, respectively. Mean total neutrophils (% CD45+ live cells gated): 10.3; 14.04; 12.37; 11.86, and 12.05 for control, 8, 17-, 26-, and 53-day groups, respectively. Mean total DC (% CD45+ live cells gated): 2.197; 2.566; 2.964; 3.413, and 3.438 for control, 8, 17-, 26-, and 53-day groups, respectively.

Figure 5 shows the relative population of total eosinophils (A), neutrophils (B), and DC (C). An increase in eosinophils was observed at 8 days compared with the control group (*p* = 0.023). The eosinophil count fell between 8 and 26 days (*p* = 0.017). Moreover, at 8 days the neutrophil population was higher than in the control group (*p* = 0.000); it then fell between 8 and 26 days (*p* = 0.041). Increases in total DC were observed at 26 and 53 days compared to controls (*p* = 0.004 and *p* = 0.005, respectively) and at 26 days and 53 days compared to 8 days (*p* = 0.017 and 0.022, respectively).

**Figure 6:**
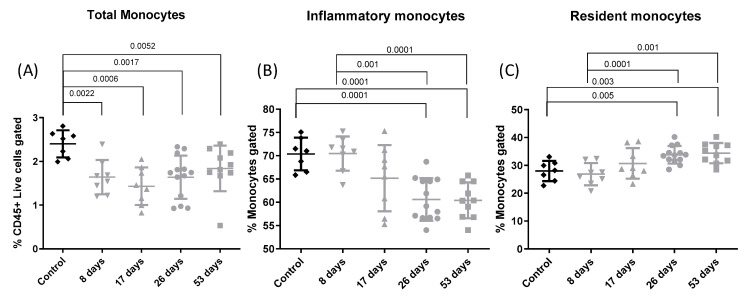
Lung total inflammatory and resident monocytes. Experimental groups are the same as in Figure 1. Individual and mean values of total (**A**), inflammatory (**B**), and resident (**C**) monocytes from flow cytometry analysis. Mean total monocytes (% MHCII cells gated): 2.402; 1.639; 1.436; 1.635; and 1.839 for control, 8, 17-, 26-, and 53-day groups, respectively. Mean inflammatory monocytes (% monocytes gated): 70.37; 70.46; 65.17; 60.6m and 60.41 for control, 8, 17-, 26-, and 53-day groups, respectively. Mean resident monocytes (% monocytes gated): 27.97; 26.84; 30.69; 33.79, and 34.36 for control, 8, 17-, 26-, and 53-day groups, respectively.

Figure 6 shows the relative populations of total (A), inflammatory (B), and resident (C) monocytes. There were decreases in total monocytes in comparison with the control group (*p* = 0.002, *p* = 0.0006, *p* = 0.0017, and *p* = 0.0052 at 8 days, 17 days, 26 days, and 53 days, respectively). Moreover, a decrease in inflammatory monocytes was observed at 26 days and 53 days compared to controls (*p* = 0.0001 in both cases) and at 26 days and 53 days compared to 8 days (*p* = 0.001 and *p* = 0.0001, respectively). An increase in resident monocytes was observed at 26 and 53 days compared to controls (*p* = 0.005 and 0.003, respectively) and at 26 days and 53 days compared to 8 days (*p* = 0.0001 and 0.001, respectively).

**Figure 7:**
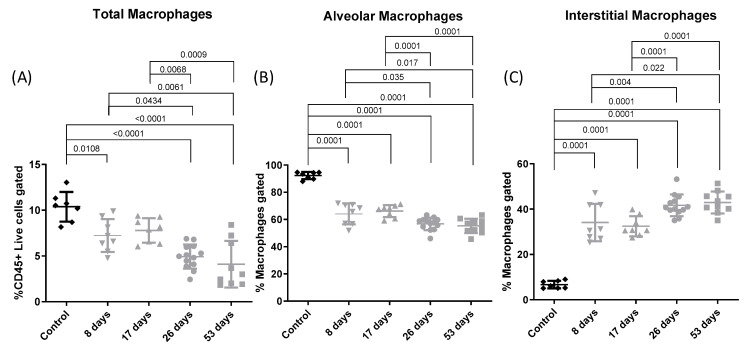
Lung total alveolar and interstitial macrophages. Experimental groups are the same as in Figure 1. Individual and mean values of total (**A**), alveolar (**B**), and interstitial (**C**) macrophages from flow cytometry analysis. Mean total macrophages (% MHCII cells gated): 65.75; 52.18; 53.7; 43.08; and 41.92 for control, 8, 17-, 26-, and 53-day groups, respectively. Mean alveolar macrophages (% macrophages gated): 92.3; 64.16; 66.18; 56.79; and 55.25 for control, 8, 17-, 26-, and 53-day groups, respectively. Mean interstitial macrophages (% macrophages gated): 6.698; 34.1; 32.45; 41.69; and 42.95 for control, 8, 17-, 26-, and 53-day groups, respectively.

Figure 7 shows the relative populations of total (A), alveolar (B), and interstitial (C) macrophages. A decrease in total macrophages was observed compared to the control group (*p* = 0.0108, *p* = 0.003, *p* = 0.0001, *p* = 0.0001 at 8 days, 17 days, 26 days, and 53 days respectively). Furthermore, a decrease in total macrophages was observed at 26 days and 53 days compared to 8 days (*p* = 0.025 and 0.022, respectively) and compared to 17 days (*p* = 0.003 and 0.005, respectively). A decrease in alveolar macrophages was observed compared to controls (*p* = 0.0001 for all groups). Therefore, further decreases were observed at 26 and 53 days compared to 8 days (*p* = 0.0035 and 0.0017, respectively) and compared to 17 days (*p* = 0.0001 in both cases). Regarding interstitial macrophages, an increase was observed compared to control group (*p* = 0.000 for all groups). Finally, increases in interstitial macrophages were observed at 26 and 53 days compared to 8 days (*p* = 0.004 and 0.022, respectively) and compared to 17 days (*p* = 0.0001 in both cases).

## 4. Discussions

In the present study the exposure of healthy mice to DEP led to a subpopulation switch in macrophages (a decrease in alveolar and an increase on interstitial macrophages) and also in monocytes (a decrease in inflammatory and an increase in resident monocytes). Increases in total neutrophils, eosinophils, and DC were also observed.

Epidemiological studies have assessed the effect of pollution on health. It has been shown that a higher concentration of particulate matter (PM) may be linked to higher mortality, due to either respiratory or cardiovascular pathologies [2]. Nevertheless, there is limited evidence on the issue of how DEP are able to affect healthy individuals. The experiment described here provides evidence of the immunomodulatory and inflammatory effects of the exposure to DEP in healthy mice.

Macrophages present a central role in clearing and processing inhaled allergens and pollutants. However, DEP have deleterious effects on macrophages, reducing their clearance capability and modulating the macrophage phenotype, generating a pro-inflammatory state [23,24]. Several in vitro studies investigated the ways in which DEP affect macrophages, exposing them to high DEP concentrations [25,26,27]. In this study we exposed macrophages to DEP in an in vivo model to determine how exposure affects healthy subjects. Exposure to DEP was reported to reduce the phagocytic capacity of alveolar macrophages and promote the expression of neutrophil chemotactic factors, which leads to a recruitment of neutrophils to the affected area [28]. In this study, an increase in neutrophils in the lumen was observed.

Moreover, a substantial increase in total loaded macrophages was observed during short and mid-term exposures, which then decreased after long-term exposure. To investigate the uptake kinetics of DEP, we further analyzed the percentage of the cytoplasmic area covered by DEP in macrophages. Our findings revealed a significant increase in DEP uptake after short- and mid-term exposure; however, after reaching this point, the percentage of area covered by DEP not only remained relatively constant but also exhibited a slight downward trend despite the continuous exposure to the particles. The findings could suggest a correlation between DEP exposure and a certain suppression of macrophage phagocytic capacity, although future studies would be necessary to corroborate this hypothesis. In this sense, the central carbon core of the DEP allows them to aggregate [11], leading to a modification in particle shape which in turn may inhibit the phagocytic process [25]. Additionally, exposure to DEP modulates the expression of specific markers in an AhR- and Nrf2-dependent manner, thereby influencing the phagocytic process and contributing to the harmful effects on the immune system [26].

Moreover, a reduction in alveolar macrophages and a rise in interstitial macrophages were observed in the study. Based on their anatomical position and function, lung macrophage subpopulations are divided into alveolar and interstitial macrophages (AM and IM, respectively) [29,30]. Alveolar macrophages may be either fetal derived tissue resident or monocyte-derived alveolar macrophages [31]. AM are the first line of defense for pathogens present in the lumen of lungs and suppress lung inflammation [32]. In steady state, they are capable of self-renewal. However, when they are depleted due to infection or injury, in order to restore the AM subpopulation, monocytes differentiate into IM [33]. In the present study, the depletion of AM during early exposure to DEP may be caused by the phagocytosis of these particles. The increase in IM is probably produced in response to the depletion of the AM population; however, further research is needed in order to define the functional response of this change.

In this study we observed a decrease in inflammatory monocytes and an increase in resident monocytes. In mice, monocytes can be inflammatory (equivalent to classical CD14++ CD16− monocytes) or resident (equivalent to non-classical CD14+ CD16+ monocytes). The exposure to DEP produced a significant decrease in both total and inflammatory monocytes but an increase in resident monocytes. In conditions of stress such as tissue damage or infection, monocytes differentiate into monocyte-derived macrophages (mo-Mac) and monocyte-derived dendritic cells (mo-DC). It has been proven that mo-Mac and mo-DC induce a pro-inflammatory state by releasing pro-inflammatory cytokines such as TNFα and IL1β [34]. After inflammation, mo-Mac play a key role in tissue repair, so disturbances in macrophage subpopulations, such as the ones caused by DEP, produce dysfunctions such as aberrant repair [35]. The recruitment of circulating resident monocytes to tissues in response to inflammation has been clearly observed in both acute [36] and chronic [37] inflammation. As far as we know, ours is the first animal model to provide evidence of the relation between exposure to DEP and enhanced eosinophilia and DC maturation, promoting a pro-inflammatory microenvironment in healthy mice. The increase in total eosinophils in the presence of pollution remains unexplored; despite some prospective studies reporting enhanced eosinophilia due to highly polluted environments in people with asthma [8,38], there are no such studies in healthy individuals.

Although innate immune cells are able to recognize and respond to pathogen-associated signals, DC primarily serve as a link between the innate and adaptive immune systems. While DC contribute to the initiation of an inflammatory response, their key function lies in processing allergen proteins and presenting them to prime naïve T cells [39]. Their appropriate maturation and optimal functioning play a crucial role in preventing the occurrence of exaggerated immune responses and autoimmune disorders. DC serve as key regulators of immune tolerance, and their proper maturation ensures a balanced immune response [40].

The present study demonstrates that continuous exposure to DEP produces a cumulative increase in DC over the exposure period. The most likely scenario is that in response to inflammation, monocytes are recruited to the damaged tissue, where they differentiate into IM during short DEP exposure and into mo-DC during long DEP exposure; both cell types present a pro-inflammatory phenotype. Enhanced DC maturation leading to an exacerbated immune response due to the presence of pollutants, has been studied in depth in vitro, and its enhancement of Th17 polarization was noted [41]. In this sense, it was demonstrated that expansion of Th17 cells contributes to DEP-mediated exacerbation of allergic asthma [42]. But the only relevant in vivo study is a prospective study performed in a cohort of 8- to 14-year-old children in the UK which also observed that the higher the concentration of pollution children were exposed to, the higher was the proportion of mature DC [9]. Those authors were even able to differentiate between DC subsets, remarking that this maturation mainly occurred in airway conventional DCs (cDC), specifically the cDC2 that efficiently promote the development of a wide range of effector CD4 T cell responses; however, as a differential marker they used CD86, which is also present in monocytes, macrophages, and mo-DC. This indicates the difficulty of differentiating between cDC2 and mo-DC [43].

Several studies have shown that most air pollutants can alter lung function parameters even in people with no previous pathologies [17,44,45] In this regard, one systematic review suggests that exposure to PM is associated with impaired lung function in adults [46]. Along the same lines, the Framingham Heart study demonstrated that short-term exposure to PM_2_._5_ is associated with lower lung function [18]. These findings are in agreement with ours, as we observed that a short exposure to DEP lowers IC and FEV0.1 in mice. These decreases are probably due to the involvement of the small airways—as can be observed by the small airway resistance and reactance. However, in our experiment mice were able to modulate their immune response and recover similar IC and FEV0.1 values for mid- and long-term DEP exposure, suggesting a compensatory effect. In any case, compensatory changes in lung volume need a few days to occur [47], which would explain why this effect is not seen after 8 days but later.

The main limitation of the present study is the administration of DEP, since the particles were applied in intranasal instillations [48] with the periodicity explained above. The main reason for this was to reduce handling (and thus animal stress), but we cannot rule out the possibility that different forms and timings of administrations could have produced different effects. Second, we used NIST SRM 2975 DEPs. These particles have a reported mean diameter by number distribution of 1.62 ± 0.01 μm, ranging from >110 μm to ultrafine particles. These DEP size equally as particles found in polluted cities. Particle sizes are relevant for respiratory analysis, as they determine effect and distribution. With these results, we cannot rule out the effect of different particle sizes. Further research is needed to characterize how different sizes would affect this situation.

## 5. Conclusions

In view of the results obtained here and of what is already known, the immunomodulatory potential of DEP is clear, as they induced a two-phase immune response in our healthy mice. The first phase, equivalent to short DEP exposure, is characterized by a decrease in lung functional parameters due to an inflammatory state mediated by neutrophilic and eosinophilic responses, affecting primarily the small airways. During this short exposure AM phagocytose DEP in order to clear both lumen and tissue. However, with high DEP accumulation AM may undergo depletion leading to neutrophilic recruitment, but also inducing a differentiation of inflammatory monocytes into IM, which could culminate in a switch of the macrophage subpopulation and make the tissue a pro-inflammatory microenvironment. In the second phase, healthy mice recover the lung function parameters with values similar to those in non-exposed mice, and there is no more eosinophilia- neutrophilia-mediated inflammation. The remaining tissue resident macrophages undergo an exhaustion process which may culminates in the loss of their phagocytic capacity. In response, DC replace macrophages in regulating the immune response, acting as key intermediators in the change from an innate to an adaptive response. All these data suggest that continuous DEP exposure triggers immune mechanisms that predispose healthy individuals to a pro-inflammatory and hyper-reactive microenvironment.

## Data Availability

The data presented in this study are available on request from the corresponding author.

## Supplementary Materials

The following supporting information can be downloaded at: https://www.mdpi.com/article/10.3390/toxics12080530/s1, Supplementary Figure S1: Tissue parameter for lung function assessment. Experimental groups are the same as in Figure 2. Individual and median values of tissue damping (A) and tissue elasticity (B). Supplementary Figure S2: Gating strategy of flow cytometric analysis from total lung homogenate. Representative contour plots and gating strategy of all analyzed cell populations by using FlowJo software. Gates containing a specific cell population are labelled with the included cell type, such as T cells; B cells; NK cells; neutrophils; eosinophils; total inflammatory and resident monocytes; total alveolar and interstitial macrophages; and total dendritic cells. Supplementary Figure S3: Experimental groups are the same as in Figure 1. Individual and mean values of total natural killers (NKs) (A), lymphocytes (B), B cells (C), and T cells (D) from flow cytometry analysis. Mean total NKs (% CD45+ Live cells gated): 6.321; 6.784; 5.983; 6.665; and 7.519 for control, 8, 17-, 26-, and 53-day groups, respectively. Mean total lymphocytes (% CD45+ live cells gated): 0.389; 0.301; 0.274; 0.553; and 0.6044 for control, 8, 17-, 26-, and 53-day groups, respectively. Mean total B cells (% CD45+ live cells gated): 23.63; 22.44; 22.89; 23.95 and 23.26 for control, 8, 17-, 26- and 53-day groups, respectively. Mean total T cells (% CD45+ live cells gated): 28.32; 26.95; 28.21; 27.39; and 27.54 for control, 8, 17-, 26-, and 53-day groups, respectively.
