# Supplementary material for: Immunological Effects of Diesel Particles in a Murine Model of Healthy Mice
Source: Toxics. 2024 Jul 23;12(8):530. doi: 10.3390/toxics12080530 (PMC11359652; doi:10.3390/toxics12080530)
Supplement: Supplementary file 1 [file toxics-12-00530-s001.zip › toxics-3100601-supplementary.pdf]

Supplementary Material. Figure S1

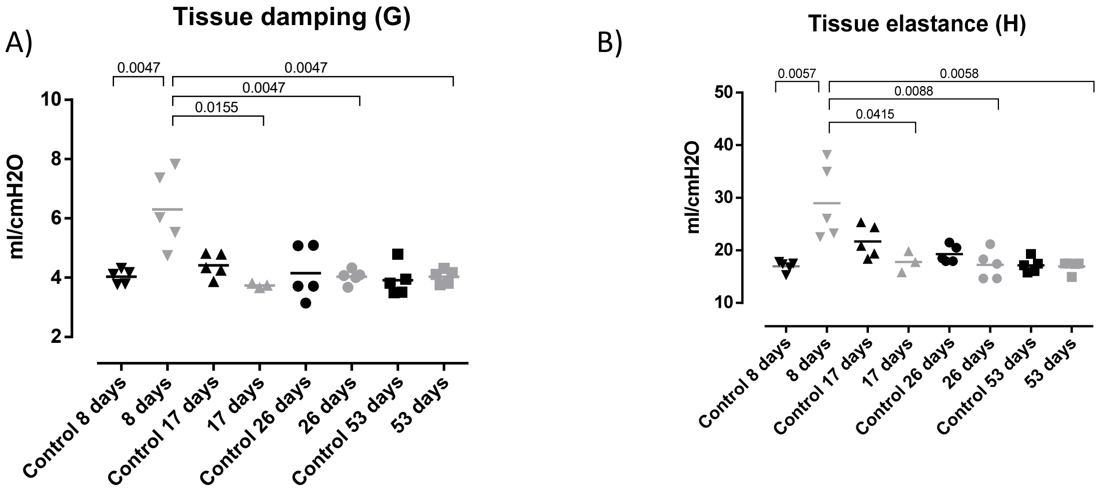

Figure S1. Tissue parameter for lung function assessment. Experimental groups are the same as in Figure 2. Individual and median values of tissue damping (A) and tissue elasticity (B).

Supplementary Material. Figure S2

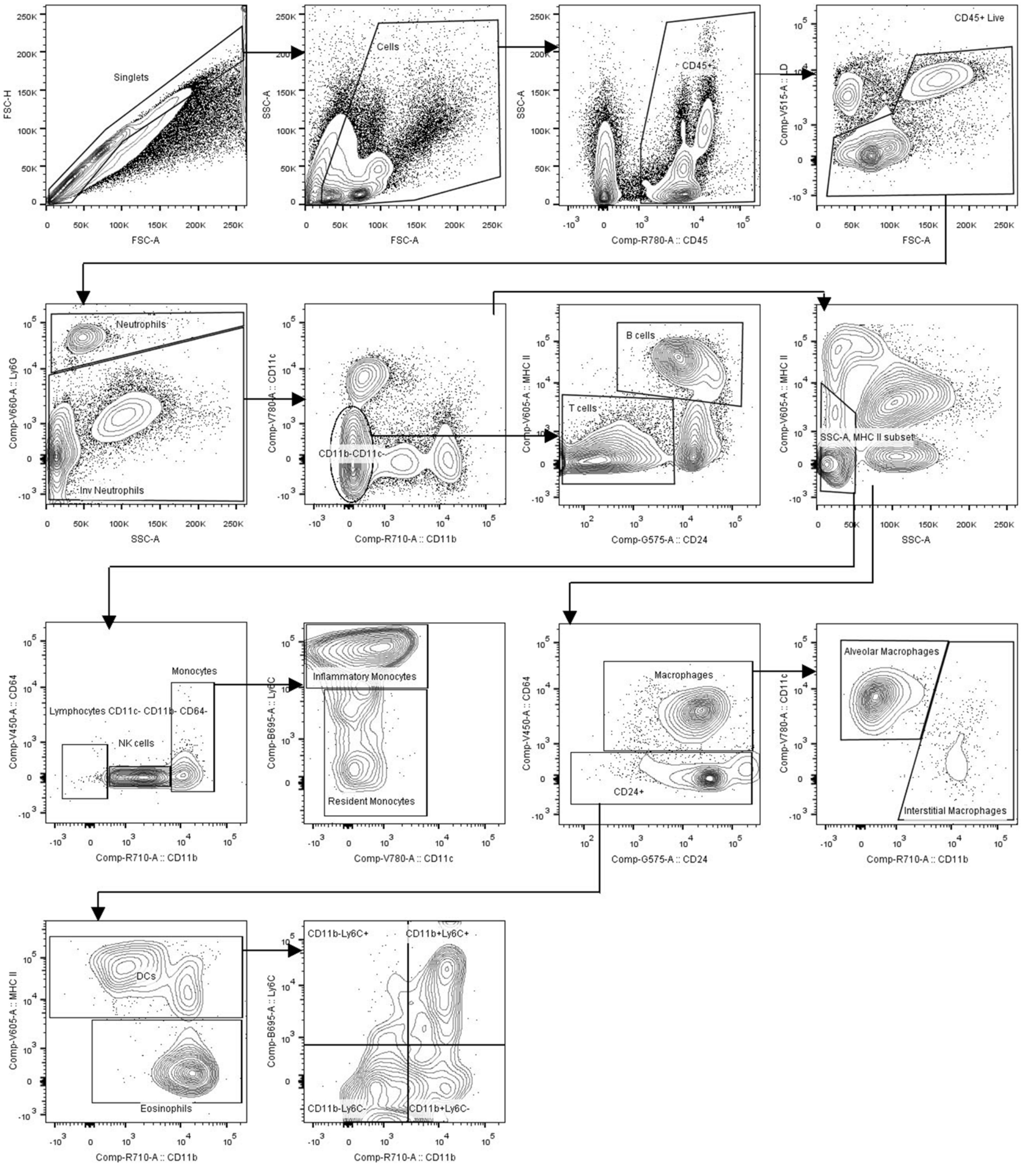

Figure S2. Gating strategy of flow cytometric analysis from total lung homogenate. Representative contour plots and gating strategy of all analyzed cell populations by using FlowJo software. Gates containing a specific cell population are labelled with the included cell type, such as T cells; B cells; NK cells; neutrophils; eosinophils; total, inflammatory and resident monocytes; total, alveolar and interstitial macrophages; and total, dendritic cells.

Supplementary Material. Figure S3

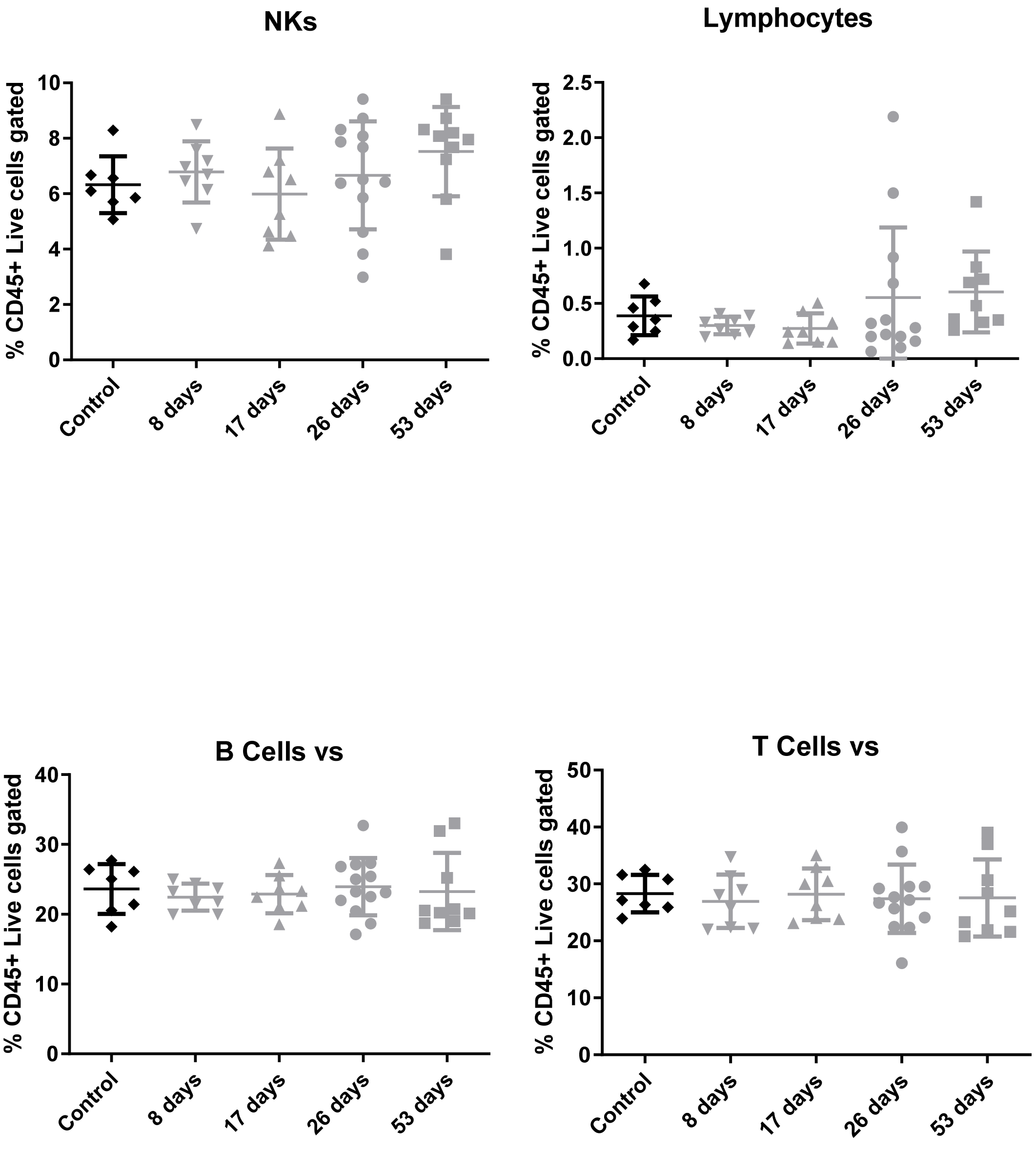

Figure S3. Experimental groups are the same as in Figure 1. Individual and mean values of total Natural Killers (NKs) (A), Lymphocytes (B), B cells (C) and T cells (D) from flow cytometry analysis. Mean total NKs (% CD45+ Live cells gated): 6,321; 6,784; 5,983; 6,665 and 7,519 for control, 8, 17-, 26- and 53-day groups, respectively. Mean total Lymphocytes (% CD45+ Live cells gated): 0,389; 0,301; 0,274; 0,553 and 0,6044 for control, 8, 17-, 26- and 53-day groups, respectively. Mean total B cells (% CD45+ Live cells gated): 23,63; 22,44; 22,89; 23,95 and 23,26 for control, 8, 17-, 26- and 53-day groups, respectively. Mean total T cells (% CD45+ Live cells gated): 28,32; 26,95; 28,21; 27,39 and 27,54 for control, 8, 17-, 26- and 53-day groups, respectively.
